# Hydrophobic Amino Acid Tryptophan Shows Promise as a Potential Absorption Enhancer for Oral Delivery of Biopharmaceuticals

**DOI:** 10.3390/pharmaceutics10040182

**Published:** 2018-10-10

**Authors:** Noriyasu Kamei, Hideyuki Tamiwa, Mari Miyata, Yuta Haruna, Koyo Matsumura, Hideyuki Ogino, Serena Hirano, Kazuhiro Higashiyama, Mariko Takeda-Morishita

**Affiliations:** Laboratory of Drug Delivery Systems, Faculty of Pharmaceutical Sciences, Kobe Gakuin University, 1-1-3 Minatojima, Chuo-ku, Kobe, Hyogo 650-8586, Japan; noriyasu@pharm.kobegakuin.ac.jp (N.K.); hideyuki.ko192@gmail.com (H.T.); poffc263@s.kobegakuin.ac.jp (M.M.); pphgl074@s.kobegakuin.ac.jp (Y.H.); pohje089@s.kobegakuin.ac.jp (K.M.); pooww214@s.kobegakuin.ac.jp (H.O.); pofip292@s.kobegakuin.ac.jp (S.H.); podtk272@s.kobegakuin.ac.jp (K.H.)

**Keywords:** tryptophan, oral delivery, insulin, GLP-1, intestinal absorption, amino acid, cell-penetrating peptide

## Abstract

Cell-penetrating peptides (CPPs) have great potential to efficiently deliver drug cargos across cell membranes without cytotoxicity. Cationic arginine and hydrophobic tryptophan have been reported to be key component amino acids for cellular internalization of CPPs. We recently found that l-arginine could increase the oral delivery of insulin in its single amino acid form. Therefore, in the present study, we evaluated the ability of another key amino acid, tryptophan, to enhance the intestinal absorption of biopharmaceuticals. We demonstrated that co-administration with l-tryptophan significantly facilitated the oral and intestinal absorption of the peptide drug insulin administered to rats. Furthermore, l-tryptophan exhibited the ability to greatly enhance the intestinal absorption of other peptide drugs such as glucagon-like peptide-1 (GLP-1), its analog Exendin-4 and macromolecular hydrophilic dextrans with molecular weights ranging from 4000 to 70,000 g/mol. However, no intermolecular interaction between insulin and l-tryptophan was observed and no toxic alterations to epithelial cellular integrity—such as changes to cell membranes, cell viability, or paracellular tight junctions—were found. This suggests that yet to be discovered inherent biological mechanisms are involved in the stimulation of insulin absorption by co-administration with l-tryptophan. These results are the first to demonstrate the significant potential of using the single amino acid l-tryptophan as an effective and versatile bioavailability enhancer for the oral delivery of biopharmaceuticals.

## 1. Introduction

During the past few decades, cell-penetrating peptides (CPPs) have been established as potential tools for delivering bioactive macromolecules and lipidic or polymeric particulate carriers into cells via mainly covalent conjugation [1,2]. Conversely, our recent studies demonstrate that CPPs can dramatically accelerate the intestinal and nasal absorption of therapeutic peptides and proteins including insulin and the GLP-1 receptor agonist, Exendin-4, by the means of noncovalent co-administration [3,4,5]. In particular, we found that artificial cationic peptides such as octaarginine (R8) and the amphipathic sequence derived from antennapedia homeoprotein—penetratin—are CPPs that have shown the greatest potential to act as bioavailability enhancers for facilitating mucosal absorption of poorly absorbable peptides and proteins [3,6]. Several reports suggested that CPPs may enter into cells by multiple internalization mechanisms including endocytosis, mainly macropinocytosis and energy-independent direct translocation into the cytoplasm compartment [7,8,9,10].

Alternatively, in an earlier study examining the effects of arginine peptides with different chain lengths (4–16 residues), Futaki and coworkers found that the cationic amino acid arginine is a key common amino acid in arginine-rich CPPs, including the human immunodeficiency virus (HIV)-1 Tat peptide and identified an optimal arginine chain length that maximized cellular internalization efficiency [11]. We also examined the relationship between chain length of arginine peptides and their ability to enhance the intestinal absorption of insulin and confirmed that the effect of R8 was stronger than that of shorter or longer peptides [12]. Apart from the discussion regarding the effects of peptides with different numbers of amino acids, we recently found that the l-form of the amino acid arginine (l-arginine) could strongly enhance the intestinal absorption of insulin in its single amino acid form, although its effectiveness was relatively less than peptide forms of arginine [13]. Other studies suggest that when hydrophobic moieties such as stearyl groups or hydrophobic amino acids such as tryptophan are found within the peptide structure they synergistically enhance the potential of the original arginine-rich peptides [14,15,16,17]. R8 modified with a stearyl group and penetratin containing tryptophan residues are typical examples of CPPs with both cationic and amphipathic properties [18,19,20]. In fact, we demonstrated the stronger effect of penetratin over R8 on the intestinal and nasal absorption of various peptide and protein drugs [3,6,21,22]. These findings have shown the importance of the hydrophobic amino acid tryptophan for boosting the action of arginine-rich peptides in the facilitation of the intestinal absorption of insulin. It was expected that single amino acid tryptophan would affect the modulation of intestinal absorption of insulin similar to l-arginine. However, little is known about the ability of tryptophan to enhance the absorption of insulin.

Therefore, in the present study, we examined the effects of single amino acid tryptophan on the intestinal absorption of insulin and compared its effectiveness to other useful and conventional absorption enhancers including CPP (penetratin), tight junction-modulator (sodium caprate, C10) [23,24,25] and lipid membrane-disrupting bile acid (sodium taurodeoxycholate) [26,27]. Among the diversity of biopharmaceuticals currently available, the majority include peptides and proteins that must be administered as injectable forms [28]. Therefore, we further evaluated the applicability of tryptophan to enhance intestinal absorption of other biopharmaceuticals including peptide drugs (GLP-1 and Exendin-4) and different sizes of model hydrophilic macromolecules (dextrans, FD-4, FD-20 and FD-70; with molecular weights of 4000, 20,000 and 70,000 g/mol, respectively). Regarding the safe use of tryptophan as a potential absorption enhancer, we evaluated the irreversibility of action and possible cytotoxic effects on the intestinal mucosa. Furthermore, to better understand the mechanism of action, the structural isomeric difference and its specificity among hydrophobic amino acids on the ability of tryptophan and the possibility of intermolecular interaction between insulin and tryptophan in absorption enhancement were examined. As the possible transepithelial pathway utilized by tryptophan, the involvement of paracellular transport via tight junction opening effect of tryptophan itself or its major metabolite serotonin was examined. In addition, the ability of tryptophan to protect the peptide drugs from intestinal enzymatic degradation was tested.

## 2. Materials and Methods

### 2.1. Materials

Recombinant human insulin (27.5 IU/mg) and amino acids (l-tryptophan, d-tryptophan, l-phenylalanine, l-isoleucine and l-proline) were purchased from Wako Pure Chemical Industries, Ltd. (Osaka, Japan). Exendin-4 was synthesized by Sigma Genosys, Life Science Division of Sigma-Aldrich Japan Co. (Hokkaido, Japan). l-Penetratin (RQIKIWFQNRRMKWKK; capital letters indicate the l-form of amino acids) and GLP-1 were synthesized by Toray Research Center, Inc. (Tokyo, Japan). d-R8 (rrrrrrrr; lowercase letters indicate the d-form of amino acids) was synthesized by Scrum Inc. (Tokyo, Japan). Fluorescein isothiocyanate-labeled dextran with average molecular weights 4400, 20,000 and 70,000 g/mol (FD-4, FD-20 and FD-70, respectively), sodium taurodeoxycholate and Triton X-100 were purchased from Sigma-Aldrich Co. (Darmstadt, Germany). C10 was purchased from Tokyo Chemical Industry, Co. Ltd. (Tokyo, Japan). Methylcellurose (MC, METOLOSE) was purchased from Shin-Etsu Chemical Co., Ltd. (Tokyo, Japan). Human colon adenocarcinoma-derived Caco-2 cell line was purchased form the American Type Culture Collection (Rockville, MD, USA) at passage 18. Dulbecco’s modified Eagle’s medium (DMEM) with 4.5 g/L glucose, nonessential amino acids (NEAA), antibiotic mixture (10,000 U/mL penicillin, 10,000 μg/mL streptomycin and 29.2 mg/mL l-glutamine in 10 mM citric acid-buffered saline), 0.05% trypsin-ethylenediaminetetraacetic acid (EDTA) and Hanks’ balanced salt solution (HBSS) were purchased from Gibco Laboratories (Lenexa, KS, USA). Fetal bovine serum (FBS) was purchased from Biowest (Nuaillé, France). Carboxymethyl dextran (CM5)-coated sensor chips were purchased from GE Healthcare (Little Chalfont, Buckinghamshire, UK). All other chemicals were of analytical grade and are commercially available.

### 2.2. Preparation of Mixed Solutions of Peptide Drugs and Amino Acids or CPPs

To prepare the insulin solution, specific amounts of recombinant human insulin were dissolved in 100 μL of 0.1 M HCl. The insulin solution was diluted with 0.8 mL of phosphate buffered saline (PBS, pH 7.4) (137 mM NaCl, 2.7 mM KCl, 8.1 mM Na_2_HPO_4_ and 1.47 KH_2_PO_4_ without calcium and magnesium) containing 0.001% MC, which prevents the adsorption of insulin and penetratin to the tube surface and was then normalized with 100 μL of 0.1 M NaOH. The final insulin concentration of the stock solution was 40 IU/mL. Stock solutions of other peptide drugs (GLP-1 and Exendin-4) and macromolecular compounds (FD-4, FD-20 and FD-70) were created by dissolving in PBS (pH 7.4) containing 0.001% MC (1.0 mg/mL and 8.0 mg/mL, respectively). Two times concentrations of amino acids (l-arginine, l- or d-tryptophan, l-phenylalanine, l-isoleucine and l-proline) were prepared using PBS (pH 7.4) containing 0.001% MC. To aid in the dissolution of high concentrations of hydrophobic amino acids such as l- or d-tryptophan and l-phenylalanine, the solutions were immersed in a water bath at 60 °C resulting in a clear solution. To create the solution used for the intestinal absorption study, two times concentrations of peptide drugs or macromolecular compounds and amino acid stock solutions were mixed together in equal volumes.

In the comparison study, specific amounts of l-penetratin, d-R8, C10, or sodium taurodeoxycholate were dissolved in PBS (pH 6.0 or 7.4) containing 0.001% MC at appropriate concentrations. For the in vitro study with Caco-2 cells, HBSS (LDH assay and transepithelial electrical resistance [TEER] measurement) or completed DMEM (WST-8 assay) were used instead of PBS to produce the test solution.

### 2.3. Animal Study

Animal experiments were performed at Kobe Gakuin University and complied with the regulations of the Committee on Ethics in the Care and Use of Laboratory Animals (approved as A16-15 and A17-4). Male Sprague Dawley rats weighing 180–220 g and male ddY mice weighing 30–40 g were purchased from Japan SLC, Inc. (Shizuoka, Japan), housed in temperature controlled rooms (23 ± 1 °C) with a relative humidity of 55 ± 5% and had free access to water and food during acclimatization. Animals were fasted for 18 h (rats) and 24 h (mice) before the experiments; however, they were allowed to drink water ad libitum.

### 2.4. In Situ Closed Ileum Loop Administration Study

Following anesthetization by intraperitoneal (i.p.) injection of sodium pentobarbital (50 mg/kg, Somnopentyl^®^, Kyoritsu Seiyaku Corp., Tokyo, Japan), which has been conventionally used in our insulin delivery study as confirmed to have a minimal effect on the blood glucose levels, rats were restrained in a supine position on a thermostatically controlled board at 37 °C. Additional i.p. injections of sodium pentobarbital (12.5 mg/kg) were administered every 1 h to maintain anesthesia. The ileum was exposed following a small midline incision made carefully in the abdomen. Its proximal to ileocecal junction segments (length = 10 cm) were cannulated at both ends using polypropylene tubing and ligated securely to prevent fluid loss. To wash out the intestinal contents, PBS warmed to 37 °C was circulated through the cannula at 5.0 mL/min for 4 min using an infusion pump. The cannulation tubing was removed, the segments were closed tightly and returned carefully to their original location inside the peritoneal cavity. Rats were kept on the board at 37 °C for an additional 30 min to calm biological reactions such as elevated blood glucose levels resulting from the stress imposed by surgery. After 30 min rest period, 0.5 mL of test insulin or other drug solution with or without amino acids and/or l-penetratin was administered directly into a 6 cm ileal loop created from the 10 cm pretreated segment. The insulin dose was administered at 50 IU/kg body weight (20 IU/mL) for all animal experiments. To examine dose dependency, the final concentrations of tryptophan were adjusted to 4, 8, 16 and 32 mM. In the comparison study with three hydrophobic amino acids or metabolite of l-tryptophan, the final concentrations of l-isoleucine, l-proline, l-phenylalanine and serotonin were 32 mM. In the study examining the synergistic effect of l-arginine and l-tryptophan, the final concentration of l-arginine was fixed at 40 mM. The doses of GLP-1 and Exendin-4 were both 1.25 mg/kg (0.5 mg/mL) and the doses of FDs were all 10 mg/kg (4 mg/mL). In the study examining the effect of pretreatment with l-tryptophan, d-R8, l-penetratin and other positive controls, ileal loop was exposed to these solutions during 30 min rest period after surgery and before the administration of insulin solution. For a part of above experiments involving pretreatment with l-tryptophan, C10 and sodium taurodeoxycholate for 30 min, an additional rest period of 30 min was added before the administration of insulin.

During the experiment, 0.25 mL blood aliquots were taken from the jugular vein at 0, 5, 10, 15, 30, 60, 120 and 180 min after dosing. The tuberculin syringes (1 mL, Terumo Corp., Tokyo, Japan) were heparinized by the standard procedure of coating the syringe wall with aspirated heparin and then expelling all the heparin. Plasma was separated by centrifugation at 13,400× *g* for 1 min and stored at −80 °C until analysis. The plasma concentrations of insulin, GLP-1 and Exendin-4 were determined using a human insulin ELISA kit (Mercodia AB, Uppsala, Sweden), GLP-1 ELISA kit (Wako Pure Chemical Industries, Ltd.) and Exendin-4 EIA kit (Phoenix Pharmaceuticals, Inc., Burlingame, CA, USA), respectively. The plasma concentrations of FDs were measured with a microplate fluorimeter (Synergy HT, BioTek Instruments Inc., Winooski, VT, USA) at excitation and emission wavelengths of 485 and 528 nm, respectively. The total area under the drug concentration time curve (AUC) from 0–3 h was estimated using the sum of successive trapezoids fitted between each set of data points in the time profiles of the plasma drug concentration.

### 2.5. Pharmacokinetic Analysis

The bioavailability of intestinal administered insulin was calculated relative to the subcutaneous (s.c.) route. Briefly, an insulin solution was prepared by dissolving an appropriate amount of insulin in PBS for s.c. injection (1 IU/kg). The peak plasma concentration (*C*_max_) and time to reach *C*_max_ (*T*_max_) were determined directly from the plasma insulin concentration–time curves. The total area under the insulin concentration–time curve (AUC) for 0–3 h was estimated from the sum of successive trapezoids between each data point. The relative bioavailability (BA) of insulin was calculated relative to the s.c. injection as follows:

BA (%) = ([AUC]/dose)/([AUC]_s.c._/dose_s.c._) × 100

### 2.6. In Vivo Oral Administration Study

After 24 h fasting, mice were dosed with insulin (50 IU/kg) with or without l-tryptophan (32 mM) (100 μL) by using oral gavage tube (i.d. 0.9 × length 50 mm) (Natsume Seisakusho Co., Ltd., Tokyo, Japan). Blood glucose levels were measured (One Touch Ultra View, Johnson & Johnson K.K., Tokyo, Japan) by collection of blood (one drop) from a small cut in the tail vein at t = 0 (prior to dosing), 15, 30, 60, 120, 180, 240, 300 and 360 min after administration. Blood glucose levels are depicted as percentage of the initial value in same mouse. Mice were trained with oral dosing and tail cutting at 24 h and 60 min prior to actual drug administration to avoid the unexpected elevation of blood glucose levels during experiments.

### 2.7. Insulin Degradation in Intestinal Enzymatic Fluid

Intestinal fluid was collected from male Sprague-Dawley rats by inserting a Sonde needle into the upper portion of the small intestine and the intestine was then cannulated on the lower side (length = 20 cm) to remove the intestinal fluid. The contents of the small intestine were washed out with 30 mL of PBS. Because intestinal fluid contains a high lipid content, the efflux solution was treated with equal volume of methylene chloride to remove any lipids that might interfere with the analysis of insulin by HPLC. This removal of lipid contents was repeated five times. l- or d-tryptophan (8–32 mM as final concentration) was mixed with insulin (10 IU/mL final concentration) and incubated in the intestinal fluid at 37 °C. At 5, 15, 30, 45 and 60 min, 50 μL was collected and added to 50 μL of ice-cold mobile phase solution to terminate the reaction. l- or d-penetratin (0.25 mM as final concentration) and STI (1.25 mg/mL final concentration) were used as a CPP and positive conventional inhibitor, respectively. Insulin concentration was measured by HPLC (LaChrom Elite System, Hitachi High-Technologies Corporation, Tokyo, Japan) using the following conditions: mobile phase, acetonitrile, trifluoroacetic acid (0.1%) and sodium chloride (31:69:0.58, *v*/*v*/*w*); 20 μL injection volume; 1.0 mL/min flow rate; 220 nm wave length; analytical column (4.6 × 150 mm; 5 μm) with guard column (4.6 × 150 mm; 5 μm) (ChemcoPak Nucleosil 5C18, Chemco Plus Scientific Co., Ltd., Osaka, Japan).

### 2.8. Cell Culture

Caco-2 cells were cultured in 75 cm^2^ culture dishes (Nippon Becton Dickinson, Tokyo, Japan) with 10 mL of culture medium. The culture medium consisted of DMEM containing 10% FBS, 0.1 mM NEAA, 2 mM glutamine, 100 U/mL penicillin and 100 μg/mL streptomycin (completed DMEM). The seeding density for cultivation was 8.0 × 10^5^ cells/dish. The cells were maintained in an incubator at 37 °C, 95% relative humidity and 5% CO_2_. The culture medium was replaced with fresh medium every second day for approximately 7 days until the cells reached 80% confluence, at which time they were subcultured. For the subculture procedure, the cells were detached from the culture dishes by trypsinization with 0.05% trypsin–EDTA, counted with a hemocytometer and transferred at the desired seeding density to new culture dishes or experimental wells. We used cells between passages 25 and 35.

### 2.9. In Vitro and In Situ the Lactate Dehydrogenase (LDH) Leakage

To assess the cytotoxic effects of l-tryptophan, the integrity of the cell membrane after treatment with the CPPs was examined by measuring the lactate dehydrogenase (LDH) released from the cytoplasm under both in vitro and in situ conditions.

In the in vitro assay, the Caco-2 cells were grown in 24-well plates with a 1.88 cm^2^ culture area (Nippon Becton Dickinson) at the density of 1.0 × 10^5^ cells/cm^2^ and then grown in completed DMEM for 3 or 4 days. Before the addition of l-tryptophan, cell membranes were allowed to equilibrate in 0.5 mL of assay buffer for 30 min. HBSS containing 0.001% MC (pH 7.4) was added to the wells as the transport buffer. After a 30 min pre-incubation with transport buffer, the assay was initiated by replacing 50 μL of transport buffer with 50 μL of l-tryptophan stock solution (ten times concentration) or transport buffer. Various concentrations (300–2400 μM) of l-tryptophan were incubated in Caco-2-seeded wells for 120 min at 37 °C. The incubation buffer was collected and LDH released from the cytoplasm into the incubation buffer was determined using a CytoTox 96 Non-Radioactive Cytotoxicity Assay kit (Promega Corp., Madison, WI, USA). Cytotoxicity was expressed as the percentage calculated by dividing the absorbance of the vehicle (HBSS) or the l-tryptophan-treated sample by that of a sample treated with 0.8% Triton X-100, according to the instructions of assay kit.

In the in situ assay, various concentrations of l-tryptophan solution (16, 32 or 50 mM) with 50 IU/kg insulin were administered into rat ileal segments in the same manner as the closed loop administration study described above. For the trial with the highest concentration of l-tryptophan (50 mM), l-arginine (40 mM) was added as a solubilizing agent. For this test, 5% sodium taurodeoxycholate was used as a positive control. At 60 min after administration, the ileal loop was washed with 5.0 mL of PBS and then intestinal fluid was collected. After adjusting the collected intestinal fluid to a total volume of 6.0 mL with PBS, the activity of LDH (Unit) leaked into the intestinal fluid was determined using an LDH kit (Promega Corp.).

### 2.10. Cell Viability Assay

To examine the cellular viability of Caco-2 cells exposed to l-tryptophan, a WST-8 assay was performed with Cell Counting Kit-8 (CCK-8, Dojindo Laboratories, Kumamoto, Japan). The Caco-2 cells were grown in 96-well plates with a 0.33 cm^2^ culture area (Nippon Becton Dickinson) to a density of 3.0 × 10^4^ cells/cm^2^ and then grown in 100 μL of completed DMEM for 3 or 4 days. In this assay, the completed DMEM was used in place of the incubation buffer to prevent the possible loss of cell viability during total 5 h incubation in HBSS. First, 10 μL of l-tryptophan stock solution (eleven times concentration) or transport buffer was added to 100 μL of completed DMEM and the cells were incubated with various concentrations of l-tryptophan (600–2400 μM) for 2 h. Then, 10 μL of CCK-8 reagent was added to the wells and the cells were incubated for an additional 3 h to allow for the reductive reaction of WST-8. The resulting absorbances of the incubated solution in the wells were read with a microplate reader (Synergy HT, BioTek Instruments Inc.) at a wavelength of 450 nm. In this assay, 0.8% of Triton X-100 was used as a positive control, same as the LDH assay.

### 2.11. Transcellular Transport Assay

For TEER measurement, Caco-2 cells were grown in 12-well transwell plates with a 1.13 cm^2^ culture area (collagen-coated polytetrafluoroethylene (PTFE) membrane with 0.4 μm pore size, Corning Inc., Corning, NY, USA). The cells were cultured to a density of 1.0 × 10^5^ cells/cm^2^. The cells were grown in completed DMEM for 21 days until they achieved a constant TEER reading of >500 Ω cm^2^, which indicated that tight junctions had formed in the monolayer. The culture medium was changed every second day and the electrical resistance was monitored by the method described below. Each well consisted of apical (top) and basal (bottom) chambers, which were separated by a collagen-coated polytetrafluoroethylene membrane with a pore size of 0.4 μm and a filter area of 1.12 cm^2^.

In preparation for the assays, the cell membranes were allowed to equilibrate for 30 min in transport buffer; HBSS (pH 7.4) with 0.001% MC was added to the apical (0.5 mL) and basal (1.5 mL) chambers of the transwell. After a 30 min pre-incubation for equilibration, an initial TEER measurement was taken of the monolayer in each well and then 100 μL of transport buffer was removed from the apical chamber and replaced with 50 μL of insulin and 50 μL of l-tryptophan or serotonin stock solutions (ten times concentration) or transport buffer. l-tryptophan (600, 1200 or 16,000 μM) or serotonin (2.4 or 16 mM) was incubated with insulin (15 μM) in the apical chamber of the Caco-2-seeded transwell for 2 h. During the incubation, the Caco-2 monolayer was kept at physiological temperature (37 °C).

### 2.12. TEER Measurement

The TEER of the Caco-2 monolayer was measured to confirm cell growth and differentiation on the insert filter of the transwell using a Millicell ERS-2 (Millipore Corp., Bedford, MA, USA). Before seeding the Caco-2 cells, the electrical resistance of the insert filter in the medium was measured and subtracted from the total electrical resistance determined for the monolayer to calculate the intrinsic TEER of the monolayer. The TEER of the monolayer was measured at the end of the 2 h incubation to detect any negative effects of the applied l-tryptophan on the intercellular tight junctions. The TEER is expressed as a percentage calculated by dividing the TEER value (Ω cm^2^) at 2 h by the initial value.

### 2.13. Surface Plasmon Resonance (SPR)-Based Binding Assay

The intermolecular interactions between insulin and l-tryptophan or l-penetratin were analyzed by SPR (Biacore X-100, GE Healthcare). To measure the binding of l-tryptophan or l-penetratin to insulin, insulin was immobilized at the carboxymethyl dextran surface of a CM5 sensor chip by using an amine coupling method. For the immobilization procedure, insulin was diluted to a concentration of 50 μg/mL using acetate buffer at pH 4.5 and immobilized on the chip surface in separate flow cells at 5 μL/min for 7 min. Reference surfaces were prepared by amine coupling activation followed by immediate deactivation. For binding measurements, different concentrations of l-tryptophan or l-penetratin (2–200 μM) were injected for 90 s followed by an additional 90 s dissociation phase. At the end of each cycle, the surface was regenerated by a 30 s injection of 1 M NaCl. Measurements were carried out in HBSS (pH 7.4) supplied with 0.001% MC at 20 μL/min at 25 °C. Each sensorgram was determined by subtracting nonspecific binding on the surface of the reference flow cell from total binding on the immobilized-insulin surface.

### 2.14. Statistical Analysis

Each value is expressed as the mean and standard error of the mean (SEM) of multiple determinations. The significance of the differences in the mean values of multiple groups was evaluated using analysis of variance with Dunnett’s test. IBM SPSS Statistics (version 24; IBM Corp., Armonk, NY, USA) was used for statistical analysis. Differences were considered significant when the *p* value was less than 0.05.

## 3. Results

### 3.1. Stimulatory Effect of l-Tryptophan on the Intestinal Absorption of Insulin

**Figure 1:**
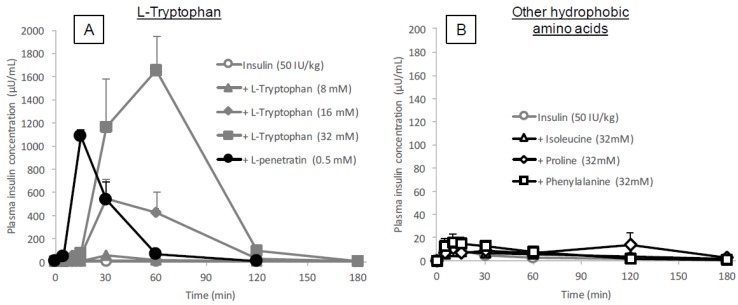
Time profiles of plasma insulin concentration after in situ administration of insulin (50 IU/kg) with or without l-tryptophan, l-penetratin or other hydrophobic amino acid additives into rat ileal loop. Panel (**A**), l-tryptophan (8–32 mM) or l-penetratin (0.5 mM); panel (**B**), hydrophobic amino acids (l-isoleucine, l-proline and l-phenylalanine, 32 mM). Each data point represents the mean ± SEM of *N* = 3–8, except for the group with l-tryptophan (8 mM, *N* = 2).

It was reported that tryptophan residues at position 6 and 14 in the penetratin amino acid sequence aid in lipid interaction in cell membranes and are therefore an important moiety involved in the cellular internalization of penetratin [18]. To test the ability of hydrophobic amino acid tryptophan as an absorption enhancer for insulin, we first conducted an in situ absorption study in which insulin was administered with various concentrations of the l-form of tryptophan (8–32 mM) into rat ileal closed loops. As shown in Figure 1A, the plasma insulin concentration increased after administration of insulin with l-tryptophan in a concentration dependent manner and with a late onset of action 30–60 min after administration. This result suggests that l-tryptophan has the potential to strongly enhance the intestinal absorption of insulin as single amino acid form. On the other hand, we confirmed the effect of the amphipathic CPP penetratin on the intestinal absorption of insulin. The effect of l-penetraitn shown in Figure 1A was consistent with our previous publication using l-penetratin [3,6], in which insulin co-administered with l-penetratin was absorbed immediately at 15 min post administration. The difference of action onset between l-tryptophan and l-penetratin suggested that l-tryptophan as single amino acid played a role in increasing absorption of insulin in a different way from tryptophan residues as a component of the peptide structure of l-penetratin. We further compared the effect of other hydrophobic amino acids such as phenylalanine, proline and isoleucine on the absorption of insulin. The results in Figure 1B show that there was no stimulatory effect on insulin absorption by these three hydrophobic amino acids suggesting that the bioenhancing effect is unique to tryptophan among the hydrophobic amino acids. The pharmacokinetic parameters are summarized in Table 1.

### 3.2. Identification of the Ability of l-Tryptophan as the Oral Absorption Enhancer for Insulin

**Figure 2:**
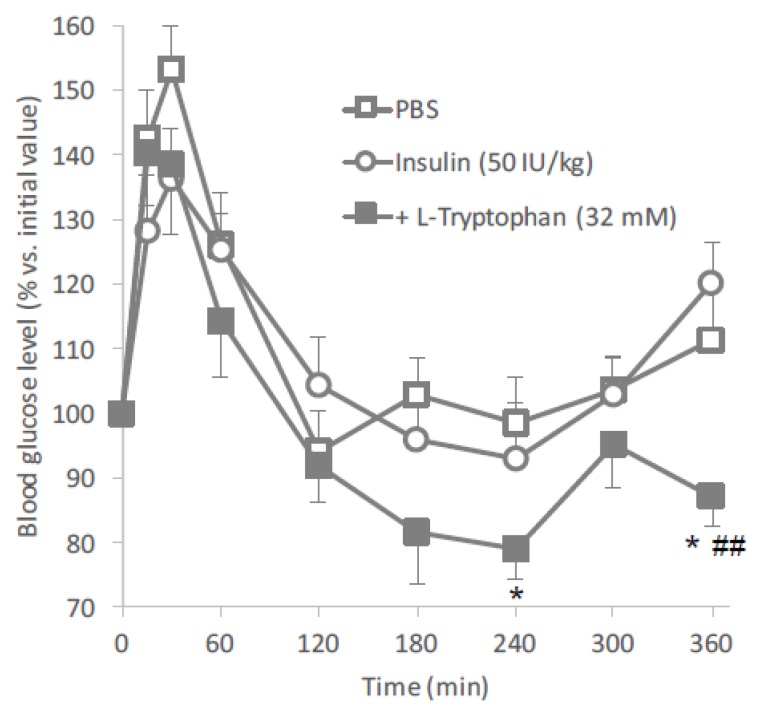
Blood glucose levels in mice following oral administration of insulin (50 IU/kg) with or without l-tryptophan (32 mM). Each data point represents the mean ± SEM of *N* = 6–8. * *p* < 0.05, ^##^ *p* < 0.01, significantly different with PBS and insulin (50 IU/kg), respectively.

The above-mentioned results suggested that l-tryptophan has the ability to effectively increase the intestinal absorption of insulin. Therefore, the potential of l-tryptophan as oral absorption enhancer was further tested after in vivo oral administration of insulin to mice. As shown in Figure 2, the hypoglycemic reaction elevated after oral administration of insulin (50 IU/kg) with l-tryptophan (32 mM) suggested that l-tryptophan could enhance the oral absorption of insulin. The elevation in the blood glucose levels just after oral administration in all groups might be because of the stress by oral gavage, even though mice were trained before administration.

### 3.3. Applicability of l-Tryptophan to Enhance the Intestinal Absorption of Various Biopharmaceuticals

Although the mechanism involved in the absorption-stimulatory effect of l-tryptophan is still unclear, our results strongly suggest that l-tryptophan has potential as an effective absorption enhancer for the oral delivery of insulin. We additionally researched the ability of l-tryptophan to aid in the delivery of other biopharmaceuticals.

**Figure 3:**
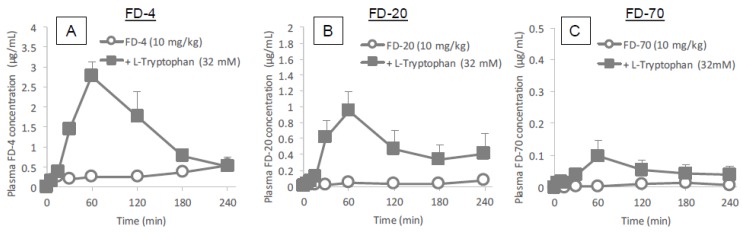
Time profiles of plasma concentrations of model hydrophilic macromolecules (FD-4, FD-20 and FD-70) after their in situ administration with or without l-tryptophan (32 mM) into rat ileal loop. Panels (**A**–**C**) show the absorption of FD-4, FD-20 and FD-70, respectively. Each data point represents the mean ± SEM of *N* = 3–8.

**Figure 4:**
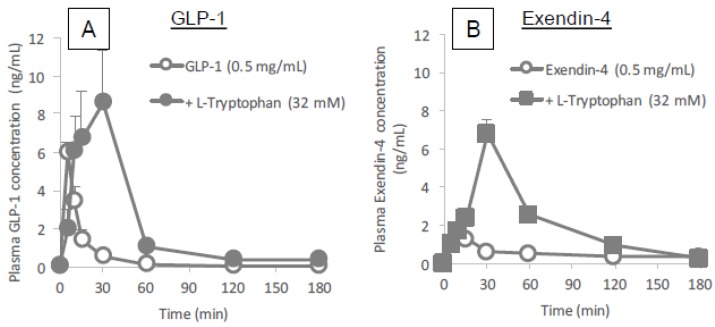
Time profiles of plasma concentrations of peptide drugs (GLP-1 and Exendin-4) after their in situ administration with or without l-tryptophan (32 mM) into rat ileal loop. Panels (**A**) and (**B**) show the absorption of GLP-1 and Exendin-4, respectively. Each data point represents the mean ± SEM of *N* = 4.

To examine the ability of l-tryptophan to assist in the transport of a range of differently sized potential drug cargo, we evaluated the effect of l-tryptophan co-administered with fluorescently labeled hydrophilic dextrans of various molecular weights (4400, 20,000 and 70,000 g/mol). As shown in Figure 3A–C, the intestinal absorption of all dextrans (FD-4, FD-20 and FD-70) was effectively enhanced by co-administration with l-tryptophan (32 mM), although the effectiveness of enhancement tended to decrease with increase in the molecular weight of the cargo compounds. Furthermore, the effect of l-tryptophan on the intestinal absorption of other peptide drugs, GLP-1 and Exendin-4, was examined. As shown in Figure 4A,B, these peptide drugs were efficiently absorbed in the presence of l-tryptophan (32 mM). Thus, l-tryptophan has the potential to enhance the intestinal absorption of a wide variety of biopharmaceuticals in a safe manner. The AUC values of dextrans and peptide drugs are summarized in Table 2.

### 3.4. Mucosal Cytotoxic Examinations for Intestinal Administration of l-Tryptophan

#### 3.4.1. Effect of l-Tryptophan on the Membrane Integrity and Cell Viability in the Intestinal Epithelium

The intestinal absorption of insulin accelerated by co-administration with l-tryptophan might be attributed to the modulation of biological structures and functions in the intestinal mucosa. To establish the safety of l-tryptophan as a potential absorption enhancer for biopharmaceuticals, changes in the integrity of epithelial cell membranes, cellular viability and the tightness of the paracellular junctions after administration of l-tryptophan were examined.

**Figure 5:**
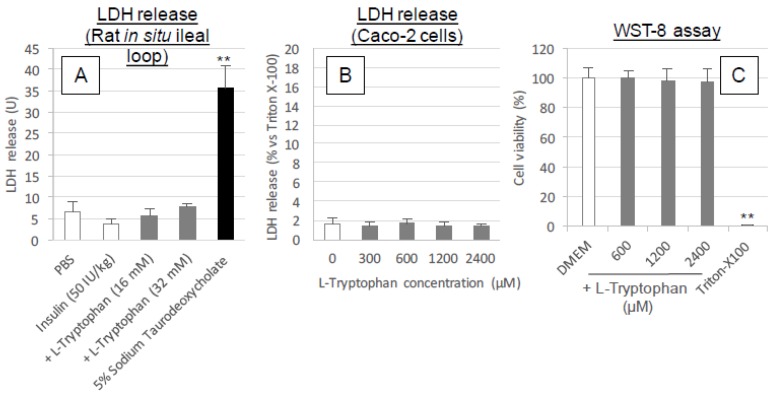
Cytotoxicity examinations after the exposure of intestinal epithelium to l-tryptophan. Panel (**A**), the LDH release in the intestinal fluid collected at 60 min after ileal administration of PBS, insulin (50 IU/kg) with or without l-tryptophan (16–50 mM) and sodium taurodeoxycholate (5%). Panel (**B**), LDH released from the cytoplasm into the incubation medium (HBSS) after incubation with various concentrations of l-tryptophan (200–2400 μM). The value is expressed as a percentage calculated by dividing the absorbance of the l-tryptophan treated medium sample by that of the sample treated with Triton X-100 (0.8%). Panel C, cell viability after incubation with various concentrations of l-tryptophan (600–2400 μM). Each data point represents the mean ± SEM of *N* = 3, 3 and 6–11 for panels (**A**–**C**), respectively. * *p* < 0.05, ** *p* < 0.01, significantly different with corresponding control PBS- (panel (**A**)), or DMEM- (panel (**C**)) treatment group.

Figure 5A,B shows the leakage of an intracellular protein, LDH, from intestinal epithelial cells after in situ administration into rat ileal loop (A) and in vitro exposure onto the epithelial model Caco-2 cells (B). In Figure 5A, the leakage of LDH was negligible after in situ administration of insulin with l-tryptophan at all tested concentrations (16 and 32 mM) and these measurements were significantly low when compared to the positive control (the administration of 5% sodium taurodeoxycholate). Similarly, in the in vitro assay (Figure 5B), no release of LDH into the media was observed after the exposure of Caco-2 cells to l-tryptophan at all concentrations (300–2400 μM). Figure 5C shows the viability of Caco-2 cells after applying l-tryptophan to culture media. The results indicate no decrease in cell viability after addition of l-tryptophan at all concentrations (600–2400 μM). The toxicity examinations suggest that l-tryptophan has no adverse effect on the intestinal mucosa and may therefore become a potentially safe absorption enhancer.

#### 3.4.2. Effect of Intestinal Pretreatment with l-Tryptophan on the Intestinal Absorption of Insulin

The results from the previous section suggest that l-tryptophan can enhance the intestinal absorption of insulin via functional and structural modification of the intestinal mucosa, while not exhibiting cytotoxicity (Figure 5). When further considering the safe use of absorption enhancers, it is important that these agents exhibit either no effect or only a temporal effect on the functions of the mucosal membrane since this membrane is an integral component of the defense system protecting against exogenous pathogens. To assess the reversibility of l-tryptophan activity, an insulin solution was administered to rat ileal loop after pretreatment with l-tryptophan (32 mM), CPPs (d-R8 and l-penetratin), or conventional enhancers (C10 and sodium taurodeoxycholate) for 30 min and then washing the pretreated ileal segment with fresh PBS.

**Figure 6:**
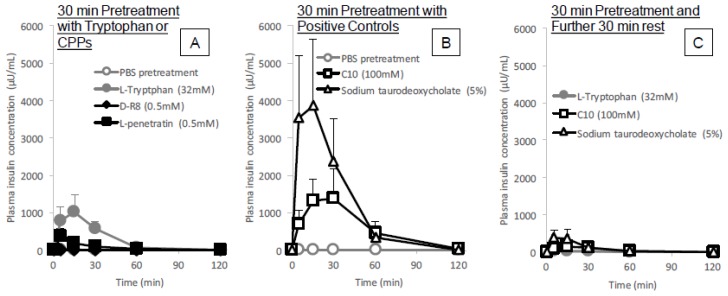
Time profiles of plasma insulin concentration after in situ administration of insulin (50 IU/kg) into rat ileal loop pretreated with l-tryptophan (32 mM), d-R8 (0.5 mM), l-penetratin (0.5 mM), C10 (100 mM), or sodium taurodeoxycholate (5%). In the results shown in Panels (**A**,**B**), insulin solution was administered immediately after washing the pretreatment solution from the ileal loop. For the results shown in Panel (**C**), insulin solution was administered at 30 min after washing out the pretreatment solution. Each data point represents the mean ± SEM of *N* = 3–4.

As shown in Figure 6A, an increase in plasma insulin concentration was observed after the administration of insulin to the ileal loop pretreated with l-tryptophan and l-penetratin, however, the extent of insulin absorption was lower than the effect of co-administration with these enhancers (Figure 1A). In addition, the onset of insulin absorption could be shifted to an early time point, (5 min after administration of insulin), by pretreating withl-tryptophan. In contrast, pretreatment with C10 and sodium taurodeoxycholate had a stronger stimulatory response on intestinal absorption of insulin than l-tryptophan and l-penetratin (Figure 6B). When insulin was administered into the ileal loop after 30 min of pretreatment with l-tryptophan followed by washing and further rest for 30 min, the increase in the plasma insulin concentration was almost completely eliminated (Figure 6C), suggesting that the action of l-tryptophan was temporal and reversible. The pharmacokinetic parameters are summarized in Table 1.

### 3.5. Mechanistic Analysis for the Intestinal Absorption of Insulin Facilitated by l-Tryptophan

#### 3.5.1. Possibility of the Intermolecular Interaction between Insulin and l-Tryptophan

The above-mentioned results suggest that single amino acid l-tryptophan is unique in its ability to enhance the intestinal absorption of insulin. The binding affinity of l-tryptophan with insulin and/or possible activity of l-tryptophan in the modulation of biological functions and structures may explain its specificity for absorption enhancement. In this section, we analyzed the intermolecular interaction between insulin and l-tryptophan using a SPR-based assay.

**Figure 7:**
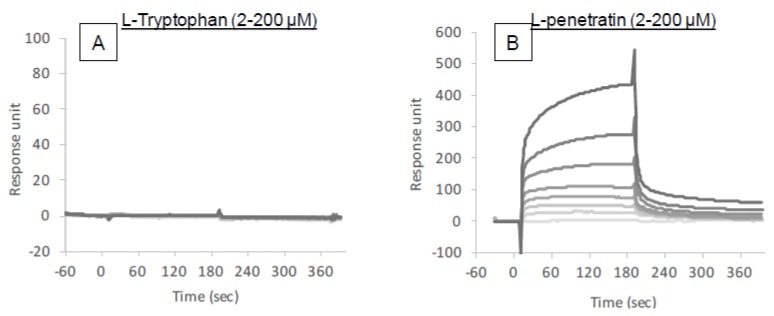
Binding sensorgrams obtained by SPR analysis. Various concentrations of l-tryptophan (**A**) or l-penetratin (**B**) solutions (pH 7.4) were injected into insulin-immobilized flow cells.

Figure 7 shows the binding sensorgrams obtained from the assay. Various concentrations of l-tryptophan (2–200 μM) or l-penetratin (2–200 μM) were injected into the flow cells containing immobilized insulin. The resulting sensorgrams do not show an increase after the injection of l-tryptophan at any concentration (Figure 7A). For comparison, l-penetratin was bound to the insulin-immobilized flow cells depending on the applied insulin concentration (Figure 7B) in a manner consistent with our previous publications [29]. These results suggest that l-tryptophan can enhance the absorption of insulin without intermolecular interaction and that the modulation of mucosal epithelial structure might be important for the enhancement of intestinal insulin absorption mediated by l-tryptophan.

#### 3.5.2. Possible Ability of Tryptophan to Protect Insulin from Enzymatic Degradation

**Figure 8:**
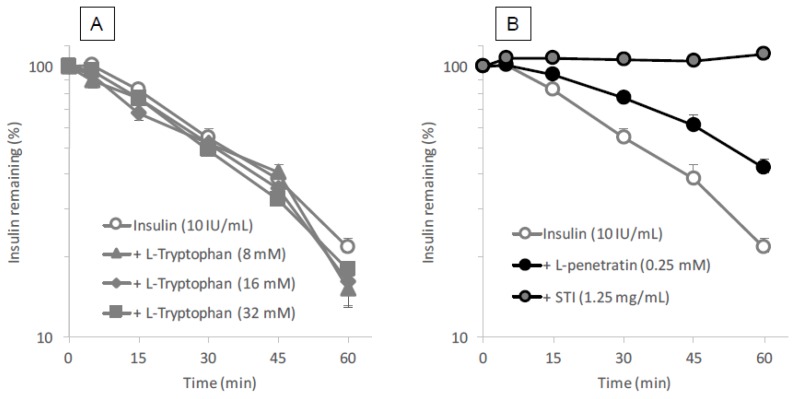
Degradation profiles of insulin in the presence of tryptophan or positive controls (penetratin and STI) in rat intestinal enzymatic fluid. Panel (**A**), various concentrations of l-tryptophan (8–32 mM); panel (**B**), l-penetratin (0.25 mM) or STI (positive control, 1.25 mg/mL). Each data point represents the mean ± SEM of *N* = 3.

The formation of the complex via intermolecular interaction between insulin and l-tryptophan or l-penetratin might be related to the protection of insulin from the intestinal enzymatic degradation. Therefore, we further evaluated the protective effect of l-tryptophan and l-penetratin on the degradation of insulin in the intestinal enzymatic fluid. As shown in Figure 8B, the positive control STI (1.25 mg/mL) completely abolished the activity of proteases in the intestinal fluid and the co-incubation with l-penetratin partially protected insulin from the enzymatic degradation. This was consistent with the result in our previous work [6]. This suggested that the stabilization of insulin in the presence of l-penetratin could be probably explained by steric hindrance via their intermolecular interaction (Figure 7B). In contrast, l-tryptophan did not have the ability to protect insulin from intestinal enzymatic degradation (Figure 8A). This may be attributed to the lack of intermolecular interaction between insulin and l-tryptophan (Figure 7A).

#### 3.5.3. Possibility of Paracellular Transport via Tight Junction Opening Effect Induced by l-Tryptophan and Its Metabolite

**Figure 9:**
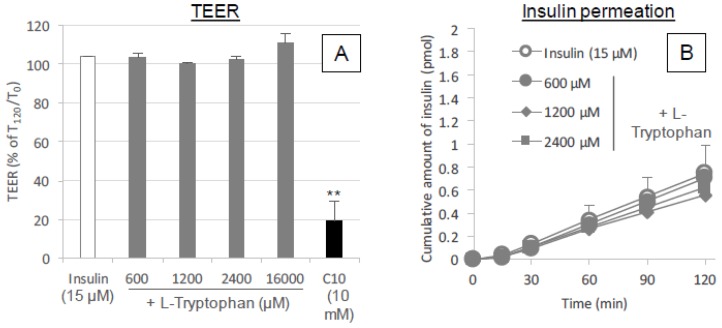
Cytotoxicity examinations after the exposure of intestinal epithelium to l-tryptophan. Panel (**A**), changes in the TEER of Caco-2 cell monolayers after the incubation with insulin (15 μM) and various concentrations of l-tryptophan (600–16,000 μM). The values are expressed as a percentage calculated by dividing the TEER measurement (Ω cm^2^) at 120 min by the initial value. Panel (**B**), time courses of permeation of insulin through Caco-2 monolayer in the presence or absence of l-Tryptophan (600–2400 μM). Each data point represents the mean ± SEM of *N* = 3. * *p* < 0.05, ** *p* < 0.01, significantly different with corresponding insulin control group.

The temporal effect of pretreatment with l-tryptophan on the intestinal absorption of insulin (Figure 6A,C) suggested that l-tryptophan could possibly modify the structure of the lipid membrane or tight junctions in the intestinal epithelium, resulting in enhanced transcellular or paracellular transport of insulin. Therefore, the epithelial transport of insulin in the in vitro Caco-2 cell monolayer model was then examined in the absence or presence of l-tryptophan. Figure 9A shows the TEER values which reflect the integrity of paracellular tight junctions after the application of l-tryptophan into Caco-2 cells grown in transwells. No change in the TEER values was observed after addition of l-tryptophan at all concentrations examined (600–16,000 μM), although a significant reduction in TEER was observed after addition of typical enhancer C10. The maintained TEER confirmed that the cellular lipid membrane was retained after addition of l-tryptophan, consistent with the LDH release assays (Figure 5A,B). Consistent with the result in TEER measurement, Figure 9B showed no change in the in vitro epithelial permeation of insulin after co-incubation with various concentrations of l-tryptophan.

#### 3.5.4. Isomeric Dependent Effect of Tryptophan Co-Administration on the Intestinal Absorption of Insulin

**Figure 10:**
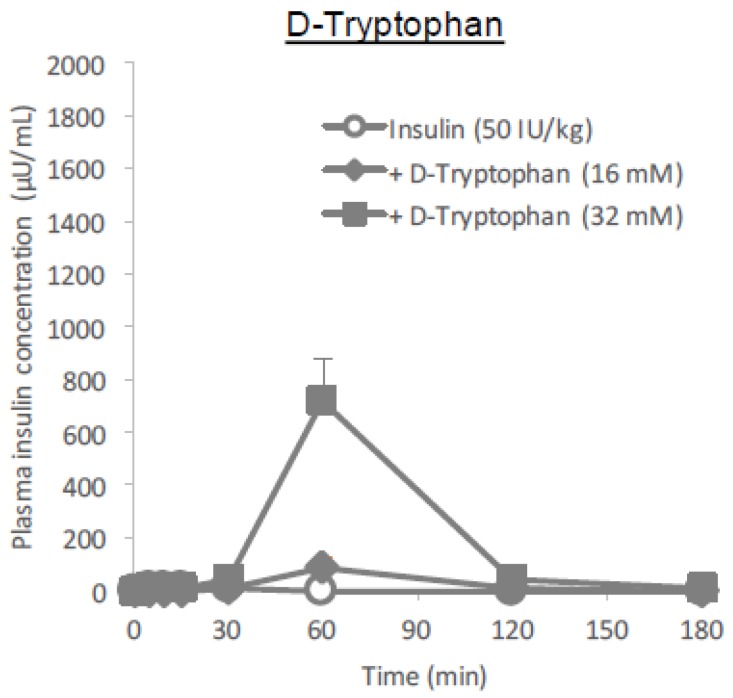
Time profiles of plasma insulin concentration after in situ administration of insulin (50 IU/kg) with or without d-tryptophan (16 or 32 mM) into rat ileal loop. Each data point represents the mean ± SEM of *N* = 3.

The lack of l-tryptophan effect in the in vitro transport assay (Figure 9B) contradicted the in situ and in vivo effect of l-tryptophan on the absorption of insulin (Figure 1A and Figure 2), suggesting that inherent active transport systems might potentially be associated with the action of l-tryptophan but that these transport systems might be diminished or deficient in Caco-2 cells. In our recent work, we found that only the l-form of arginine in its amino acid form can facilitate the intestinal absorption of insulin [13]. As it is possible that the effect of tryptophan may also vary between l- and d-forms, we conducted an in situ absorption study of insulin with d-tryptophan (16 and 32 mM) and compared its effectiveness to that of l-tryptophan (Figure 1A). The results in Figure 10 show that the effect of d-tryptophan appears much weaker than that of l-tryptophan but the onset of action by d-tryptophan (60 min after administration) was similar to that of l-tryptophan.

#### 3.5.5. Possible Involvement of Serotonin as Active Metabolite of l-Tryptophan to Enhance the Intestinal Absorption of Insulin

**Figure 11:**
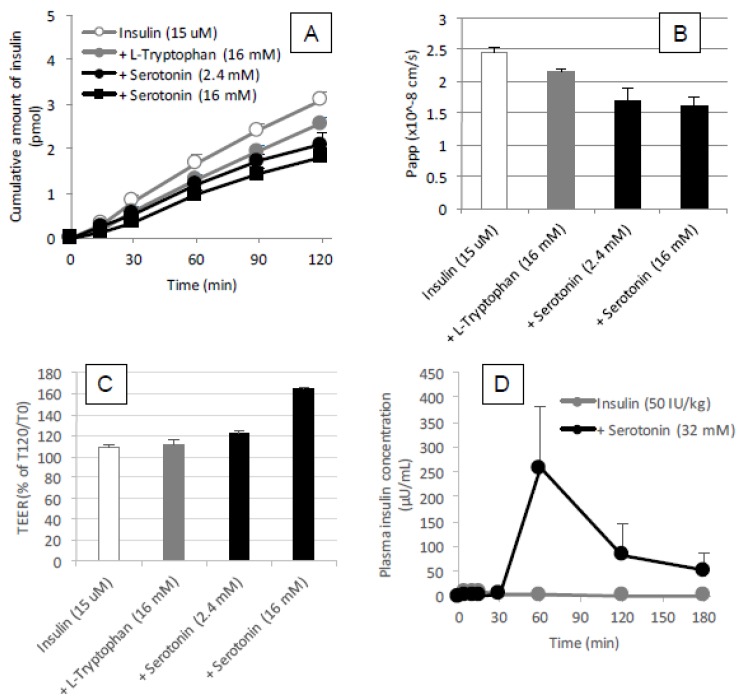
Permeation of insulin through the Caco-2 cell monolayer panels (**A**,**B**) and TEER values panel (**C**) during coincubation with l-tryptophan (16 mM) or serotonin (2.4 or 16 mM) and the absorption of insulin after its in situ administration of insulin (50 IU/kg) with or without serotonin (32 mM) into rat ileal loop panel (**D**). Each data point represents the mean ± SEM of *N* = 3.

On the other hand, it is possible that the metabolite of l-tryptophan, serotonin, has the potential to enhance the epithelial permeability of insulin and other macromolecular drugs [30] and the delayed action of l-tryptophan might be attributed to the time required for conversion from tryptophan to serotonin after administration to the intestine. Therefore, we then tested the effect of serotonin on the permeation and absorption of insulin under the in vitro and in situ conditions. The result of the permeation study with Caco-2 cell monolayer (Figure 11A,B) showed that serotonin had no stimulatory effect on the permeation of insulin, in fact, serotonin increased the TEER as shown in Figure 11C, suggesting that paracellular space was tightened by addition of serotonin. Consistent with the increase in TEER, the permeability of insulin through the Caco-2 cell monolayer decreased by co-incubation with serotonin (Figure 11B). On the other hand, serotonin (32 mM) could enhance the intestinal absorption of insulin after administration to a rat ileal loop but it was less effective than l-tryptophan (Figure 11D). The pharmacokinetic parameters are summarized in Table 1. The relationship between results in the in vitro and in vivo conditions is unknown.

#### 3.5.6. Combined Effect of Co-Administration of l-Tryptophan with l-Penetratin on the Intestinal Absorption of Macromolecules

The difference in the action onset between l-tryptophan (slow) and l-penetratin (rapid) shown in Figure 1A suggested that these two enhancers used different pathways to enhance the intestinal absorption of insulin and other macromolecular drugs. No intermolecular interaction between insulin and l-tryptophan (Figure 7A,B) confirmed that l-tryptophan uses a different mechanism than l-penetratin. Therefore, we then examined the additive or synergistic potential of the combination of l-tryptophan and l-penetratin to increase the intestinal absorption of macromolecular dextran (FD-4) in the in situ ileal loop administration study.

**Figure 12:**
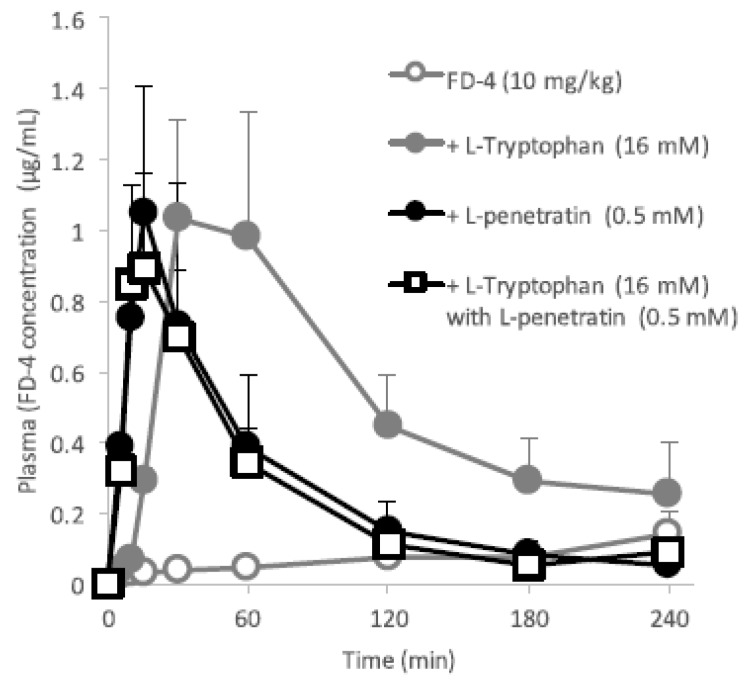
Time profiles of plasma concentrations of FD-4 after its in situ administration with or without l-tryptophan (16 mM) and/or l-penetratin (0.5 mM) into rat ileal loop. Each data point represents the mean ± SEM of *N* = 3–4.

As shown in Figure 12, the individual effect of l-tryptophan (16 mM) and l-penetratin (0.5 mM) on the absorption of FD-4 was confirmed as being delayed and rapid onset, respectively, consistent with the above-mentioned results with insulin (Figure 1). Contrary to our expectation, the effect of the combined co-administration with l-tryptophan (16 mM) and l-penetratin (0.5 mM) on the absorption of FD-4 was almost equal to that co-administered with only l-penetratin (0.5 mM). As l-penetratin had the capacity to interact with FD-4 similar to insulin, l-tryptophan might be deprived of a chance to facilitate the absorption of FD-4. Possibly, a large part of FD-4 administered to the intestine could be systemically and quickly absorbed by complexing with l-penetratin, while FD-4 which could be delivered by l-tryptophan was scarce due to the slow mechanism of action. The results suggested that the action mechanism of l-tryptophan to facilitate the intestinal absorption of macromolecular drugs was different from that of l-penetratin.

## 4. Discussion

We recently discovered that single amino acid arginine, particularly the l-form of arginine, an important component in cationic CPPs, has the potential to facilitate the intestinal absorption of insulin without being part of a peptide structure such as arginine-rich CPPs [13]. Another amino acid, the hydrophobic amino acid tryptophan, is known to play an important role in the cellular association and internalization of the amphipathic CPP, penetratin [16,18]. In fact, our previous study demonstrated that the amphipathic CPP enhanced the intestinal absorption of insulin to a greater degree than cationic CPPs such as R8 and Tat peptide [3]. Therefore, we hypothesized that addition of tryptophan to insulin might potentially improve the intestinal absorption of insulin.

The data showed that the intestinal absorption of insulin was significantly enhanced by co-administration with l-tryptophan (16 or 32 mM, Figure 1A and Table 1), comparable to the effect of l-penetratin (0.5 mM). Interestingly, the onset patterns of insulin absorption after co-administration were quite different between l-arginine (*T*_max_ 12.8 ± 0.9 min at 40 mM) and l-tryptophan (*T*_max_ 52.5 ± 7.5 min at 32 mM), as shown in Figure 1A, Table 1 and our previous publication [13], suggesting that different mechanisms might be associated with the absorption enhancement by cationic arginine versus hydrophobic tryptophan. On the other hand, we showed in our previous publications that the simple cationic CPP (R8) generated a relatively slow enhancing effect, whereas the typical amphipathic CPP (penetratin), containing two tryptophan residues, rapidly enhanced the absorption of insulin [3,21], contradicting the effect of amino acids, l-arginine and l-tryptophan, observed in the present and current studies [13]. This suggests that l-tryptophan and l-arginine could enhance intestinal absorption via mechanisms distinctly different from that involving CPPs.

The binding analysis showed no intermolecular interaction between insulin and l-tryptophan (Figure 7), implying that l-tryptophan has no capacity to bring the noncovalently mixed drugs as a delivery carrier. l-arginine also showed no binding with insulin (data not shown). This observation is quite different from the mechanism of penetratin and R8 in which intermolecular interaction is essential to their ability as absorption enhancers. There was no protective effect of l-tryptophan on the enzymatic degradation of insulin (Figure 8A), confirming no possible interaction between insulin and l-tryptophan. With respect to the opening of tight junctions as a possible mechanism, the examination in our recent report suggested that no opening of tight junctions was observed after adding the effective concentrations of l-arginine (9.6 mM equivalent to 4.2 mg/kg in the animal study) to the transwell with Caco-2 cell monolayers [13]. In regard to tryptophan, while there is an earlier report suggesting that mucosal exposure of l-tryptophan (1 mM or more) increases the paracellular permeability of macromolecules in an energy- and sodium-dependent manner [31], more recent study suggests that indole-containing molecules like tryptophan strengthen the epithelial barrier function via the increased expression of tight junction-related proteins such as claudins and occludins [32,33]. No change in the TEER values of Caco-2 cell monolayers was observed after addition of various concentrations of l-tryptophan (600–16,000 μM) (Figure 9A). Therefore, it is expected that the mechanism resulting in the enhancement of the mucosal absorption by both amino acids does not occur via opening of tight junctions.

Interestingly, it was suggested that the enteric nervous system was associated with the intestinal transport of low molecular weight compounds and hydrophilic macromolecules [34,35]. While the adrenergic stimulation has been known to decrease the intestinal absorption of dextran with molecular weights of 20,000 and 40,000 g/mol [35], vascular perfusion with the cholinergic agonist bethanechol can contribute to the increase in intestinal drug transport [34]. Furthermore, serotonin, a major metabolite of tryptophan, is known to promote the secretion of acetylcholine via the stimulation of the serotonin 5-HT_4_ receptor, possibly contributing to the increased intestinal absorption of macromolecules [30]. No enhancement of the epithelial permeation of insulin in the presence of l-tryptophan (Figure 9B) may strengthen the hypothesis that l-tryptophan needs to be metabolized for enhancing the epithelial permeation of drugs. However, our in vivo data (Figure 11D) in the present study was inconsistent with such a hypothesis, as the effect of serotonin (32 mM) on the intestinal absorption of insulin was weaker than that of l-tryptophan (32 mM) and the action onset of serotonin was delayed compared to l-tryptophan. Furthermore, our in vitro data partially negated the possible cholinergic contribution, as serotonin appeared to tighten the paracellular space and decrease the epithelial transport of insulin through a Caco-2 cell monolayer (Figure 11A–C). In the present study regarding the ability of tryptophan to enhance intestinal absorption, the l-form is more effective than the d-form (Figure 10), similar to arginine. Considering the differences between how l- and d-amino acids affect insulin absorption, endogenous systems such as the enteric nervous system, the transporter-mediated influx, or receptor-mediated influx mechanisms might be involved either directly or indirectly. Although some effects of d-tryptophan remained at the higher dose (32 mM), it was considerably small compared to that of l-tryptophan (Figure 10 and Table 1), suggesting that stereospecificity might be partially involved in their absorption enhancing mechanisms.

The in situ administration study conducted after pretreatment with l-tryptophan indicated its reversibility in the enhancement of the intestinal absorption of insulin. As shown in Figure 6C, the absorption stimulatory effect of l-tryptophan greatly diminishes at 30 min after pretreatment with l-tryptophan followed by washing, like the effect of conventional positive controls such as C10 and sodium taurodeoxycholate. These results along with the cytotoxic analysis (Figure 5A–C and Figure 9A) suggest l-tryptophan has no irreversible effect on the mucosal structure, which means that l-tryptophan does not induce permanent functional change to the intestinal membrane. Future studies are needed to clarify the mechanism.

Based on the results of the in situ intestinal insulin absorption study, we examined the potential of l-tryptophan as an oral absorption enhancer for insulin in vivo. Figure 2 clearly showed the hypoglycemic effects after oral administration of insulin (50 IU/kg) with l-tryptophan (32 mM). In this study, insulin and l-tryptophan were administered just as a solution, therefore, the development of an appropriate formulation could be expected to increase the biological effect of insulin. Figure 3 and Figure 4 clearly demonstrates the wide applicability of l-tryptophan as an absorption enhancer for macromolecules having peptide- to protein-sized molecular weights. In contrast, l-arginine had no effect on the intestinal absorption of such peptide drugs (data not shown). The effectiveness of absorption enhancement by l-tryptophan was comparable to that by l-penetratin, although l-tryptophan required relatively higher concentrations (16 mM or more) than l-penetratin (0.5 mM). Since l-tryptophan is currently used as an oral supplement and it appears to facilitate the intestinal absorption of a wide variety of biopharmaceuticals, its potential as an intestinal absorption enhancer is meritorious and quite unique.

As mentioned above, tryptophan is first metabolized into serotonin which stimulates the cholinergic nervous system, balancing neural activity. Therefore, although l-tryptophan is one of the essential amino acids, its excess use has to be carefully considered for safe pharmacotherapy. In addition, another metabolite from tryptophan, melatonin, is used as a sleep-inducing agent and therefore may affect circadian rhythm. The required dose of l-tryptophan for inducing sleepiness is roughly to 1500–3000 mg·day^−1^·human^−1^, which is comparable to the maximum dose used to enhance the intestinal absorption of biopharmaceuticals (32 mM equivalent to 16.3 mg/kg) in this study. However, the dose of l-tryptophan used as an absorption enhancer could be further optimized by precisely evaluating the relation between its co-administered concentration and the efficiency of the resultant absorption enhancement. Considering the effective plasma concentration of insulin (<100 μU/mL), it is expected that the dose of l-tryptophan can be reduced to 10 mM, the equivalent to 5.1 mg/kg (300 mg/human). Further examination will be essential to establish the potential of l-tryptophan as a safe and effective absorption enhancer for the oral delivery of biopharmaceuticals.

## 5. Conclusions

In the process of studying the role of the hydrophobic amino acid tryptophan, which was widely known as a key amino acid in the function of CPPs, we discovered that tryptophan singly improves the intestinal absorption of various biopharmaceuticals having peptide to protein molecular sizes. When examining the absorption of insulin in particular, the bioavailability enhancement effect of l-tryptophan was far stronger than that of l-arginine [13], another key cationic amino acid component of CPPs and comparable to the potent CPP, l-penetratin. However, the mechanism of action remains unclear. In the present study, the cell integrity of the intestinal epithelium (demonstrated by assessing cell viability, cell membrane integrity and the tightness of paracellular junctions) was not changed in the presence of l-tryptophan. Furthermore, no intermolecular interaction between insulin and l-tryptophan was observed and insulin was not protected from enzymatic degradation when in the presence of l-tryptophan. Tryptophan is the precursor of a wide array of bioactive compounds, such as serotonin, melatonin and so forth. [36]. Considering the stronger action of l-tryptophan as an absorption enhancer relative to the d-form, inherent biological functions might be involved in its action mechanisms. To utilize l-tryptophan as a safe absorption enhancer, further studies are required to identify the complete mechanism of action.

## Figures and Tables

**Table 1:** Pharmacokinetic parameters following in situ administration of insulin with various amino acid additives, l-penetratin, or after pretreatment of amino acids or control reagents.

			Experiment	*C*_max_ (μU/mL)	*T*_max_ (min)	AUC (μU·h/mL)	BA (%)
Insulin (50 IU/kg)			8	10.6 ± 2.4	9.4 ± 1.1	8.5 ± 2.5	0.1 ± 0.0
	+l-Tryptophan	(8 mM)	2	52.9	30	35.8	0.4
		(16 mM)	4	563.0 ± 162.4	45.0 ± 8.7	558.4 ± 195.2 **	5.8 ± 2.0 **
		(32 mM)	4	2008.2 ± 196.9	52.5 ± 7.5	1785.7 ± 153.5 **	18.7 ± 1.6 **
	+d-Tryptophan	(16 mM)	3	84.7 ± 35.8	60.0 ± 0.0	75.4 ± 30.2	0.8 ± 0.3
		(32 mM)	3	723.5 ± 153.0	60.0 ± 0.0	606.3 ± 132.8 **	6.3 ± 1.4 **
	+l-penetratin (0.5 mM)		4	1079.0 ± 54.4	15.0 ± 0.0	481.8 ± 85.0 **	5.0 ± 0.9 **
	+Serotonin (32 mM)		3	258.3 ± 124.7	60.0 ± 0.0	304.8 ± 174.1	3.2 ± 1.8
Pretreatment study							
	+PBS		4	1.7 ± 0.3	7.5 ± 2.5	2.0 ± 0.2	0.0 ± 0.0
	+l-Tryptophan (32 mM)		4	1040.5 ± 455.9	18.8 ± 3.8	582.1 ± 229.2	6.1 ± 2.4
	+d-R8 (0.5 mM)		3	22.7 ± 5.2	8.3 ± 3.3	22.5 ± 6.7	0.2 ± 0.1
	+l-penetratin (0.5 mM)		3	356.3 ± 241.3	5.0 ± 0.0	141.0 ± 73.8	1.5 ± 0.8
	+C10 (100 mM)		3	1574.8 ± 772.9	20.0 ± 5.0	1235.9 ± 686.2	12.9 ± 7.2
	+Sodium taurodeoxycholate (5%)		3	4038.0 ± 1685.4	8.3 ± 3.3	2386.2 ± 1114.6 *	25.0 ± 11.7 *
Pretreatment with 30 min rest							
	+l-Tryptophan (32 mM)		3	41.2 ± 11.2	11.7 ± 3.3	25.8 ± 5.4	0.3 ± 0.1
	+C10 (100 mM)		3	165.1 ± 22.7	11.7 ± 3.3	128.3 ± 35.6	1.3 ± 0.4
	+ Sodium taurodeoxycholate (5%)		3	393.7 ± 225.2	8.3 ± 3.3	203.3 ± 121.3	2.1 ± 1.3
Insulin s.c. (1 IU/kg)			3	133.8 ± 3.4	25.0 ± 5.0	191.2 ± 5.2	100

*C*_max_, the maximum concentration; *T*_max_, the time to reach the *C*_max_; AUC, the area under the curve; BA, relative bioavailability compared to subcutaneous injection. Each data point represents the mean ± SEM of *N* =3–8, except for the group with l-tryptophan (8 mM, *N* = 2). * *p* < 0.05, ** *p* < 0.01, significantly different with corresponding control insulin (50 IU/kg).

**Table 2:** AUCs following in situ administration of dextrans (FD-4, FD-20 and FD-70) with l-tryptophan into rat ileal loop.

	AUC (ng·h/mL)	
	−l-Tryptophan	+l-Tryptophan (32 mM)
FD-4 (10 mg/kg)	1194.2 ± 463.2	5512.7 ± 1034.9**
FD-20 (10 mg/kg)	146.0 ± 44.8	1973.2 ± 801.8*
FD-70 (10 mg/kg)	25.4 ± 24.6	205.6 ± 115.0
GLP-1 (0.5 mg/mL)	1.35 ± 0.27	6.35 ± 2.06*
Exendin-4 (0.5 mg/mL)	1.54 ± 0.62	6.17 ± 0.67**

AUC, the area under the curve. Each data point represents the mean ± SEM of *N* = 3–8. * *p* < 0.05, ** *p* < 0.01, significantly different with corresponding control without l-tryptophan.
